# Plasma levels and peripheral DNA methylation of IGF-1 and IGF-2 across knee osteoarthritis pain phenotypes in middle-aged and older adults

**DOI:** 10.1016/j.ynpai.2026.100233

**Published:** 2026-09-07

**Authors:** Muhammad Abbas, Javier A. Tamargo, Yutao Zhang, Carlos J. Cruz, John Gilliam, Stephanie Wohlgemuth, Kevin Wu, Roland Staud, Li Chen, Burel R. Goodin, Roger B. Fillingim, Christiaan Leeuwenburgh, Yenisel Cruz-Almeida

**Affiliations:** a Department of Community Dentistry and Behavioral Science, University of Florida, Gainesville, FL, USA; b Pain Research and Intervention Center of Excellence, University of Florida, Gainesville, FL, USA; c Department of Biostatistics, University of Florida, Gainesville, Florida, USA; d Department of Applied Physiology and Kinesiology, Laboratory for Rehabilitation Neuroscience, University of Florida, Gainesville, FL, USA; e Department of Physiology and Aging, College of Medicine, University of Florida, Gainesville, FL, USA; f Department of Medicine, College of Medicine, University of Florida, Gainesville, FL, USA; g Department of Anesthesiology, Washington University School of Medicine, St. Louis, MO, USA; h Department of Medicine, Division of Geriatric Medicine, College of Medicine, Institute on Aging, University of Florida, Gainesville, FL, USA; i Department of Neuroscience, College of Medicine, University of Florida, Gainesville, FL 32610, USA; j Center for Cognitive Aging and Memory, McKnight Brain Institute, University of Florida, Gainesville, FL, 32610, USA

**Keywords:** Insulin growth factors, Plasma protein, Epigenetics, Osteoarthritis, Pain, Older adults

## Abstract

Osteoarthritis (OA) is a leading cause of pain and disability, yet the biological mechanisms linking structural disease and pain remain incompletely understood. Insulin-like growth factors (IGFs) are involved in cartilage homeostasis, inflammation, and pain signaling and may serve as biomarkers or therapeutic targets in OA. This exploratory cross-sectional study examined associations between circulating levels and peripheral blood DNA methylation of *IGF1 and IGF2* with chronic knee OA pain phenotypes in 158 adults aged 45–85 years with and without symptomatic knee OA. Participants were classified into five radiographic OA (ROA)-pain phenotypes based on pain impact and Kellgren-Lawrence grade: no pain/no ROA, pain/no ROA, pain/late ROA, high-impact pain/early ROA, and low-impact pain/early ROA. In adjusted analyses, ROA-pain phenotypes were associated with circulating IGF-1 and IGF-2 levels in a sex-dependent manner. Females with low-impact pain and early ROA had higher IGF-1 levels than males in the same phenotype. For IGF-2, males with no ROA, with and without pain, had higher levels than males with high-impact pain and early ROA, and males with no pain/no ROA also had higher IGF-2 levels than females. Mean DNA methylation levels of *IGF1* and *IGF2* showed limited associations after covariate adjustment, and no probe-level findings survived multiple-testing correction. These findings identify sex-dependent associations between circulating IGF-1 and IGF-2 and knee OA pain phenotypes, most notably lower IGF-2 in males with high-impact pain and early radiographic disease. The largely null peripheral methylation results suggest that tissue-level epigenetic regulation of IGF pathways, beyond what the buffy coat captures, warrants further investigation in larger cohorts.

## Highlights

• Plasma IGF-1 and IGF-2 levels were associated with ROA-pain phenotype in a sex-dependent manner.
• Females with low-impact pain and early ROA had higher IGF-1 levels than males.
• Among males, plasma IGF-2 was lower with high-impact pain and early ROA.
• Mean *IGF1* and *IGF2* methylation showed limited associations with these phenotypes.
• Plasma IGF-2 showed a stronger association with ROA-pain phenotypes than IGF-1.

## 1 Introduction

Osteoarthritis (OA) is a leading cause of chronic joint pain and disability, especially in the knee (Abbas et al., 2025; Abbas et al., 2026). OA is increasingly recognized as an age-related disease involving inflammatory signaling, mitochondrial dysfunction, and epigenetic alterations within joint tissues and sensory pathways (Abbas et al., 2025; Motta et al., 2023). Among these, DNA methylation has emerged as a key regulatory mechanism in OA, influencing chondrocyte function, extracellular matrix remodeling, and inflammatory pathways underlying cartilage degeneration (Barter et al., 2012; Peffers et al., 2018; Rice et al., 2020). DNA methylation has been implicated in musculoskeletal and painful disorders through effects on nociceptor sensitivity and neuroimmune signaling, with differential methylation patterns associated with pain severity in OA and related conditions (Montesino-Goicolea et al., 2022; Descalzi et al., 2015; Liang et al., 2015; Xiong et al., 2024). In knee OA, chronic pain arises from complex biopsychosocial processes including bidirectional loops between nociception and progressive tissue degeneration (Zhai and Huang, 2024; Knights et al., 2023).

Insulin-like growth factor (IGF) signaling is a key regulator of joint homeostasis and a promising therapeutic target in OA, as it may simultaneously alleviate pain and promote joint tissue regeneration (Wen et al., 2021; Ma et al., 2023; Uchimura et al., 2015; Cho et al., 2025). Systemically, IGF-1 signaling, which declines with aging, promotes collagen and other extracellular matrix (ECM) components, supports chondrocyte survival, and inhibits cytokine-mediated catabolic pathways (Atwal et al., 2023; Dixit et al., 2021). IGF-2 has also been implicated in OA-related joint health, with evidence that it can prevent osteophyte growth and subchondral bone sclerosis in vivo, and preserve cartilage integrity (Uchimura et al., 2015). IGF-2 is of particular interest due to its regulation through genomic imprinting (Ferguson-Smith, 2011; Nordin et al., 2014), highlighting a potential intersection between IGF signaling and age-related DNA methylation changes relevant to OA pain.

IGF activity is regulated by IGF-binding proteins (IGFBPs), which regulate ligand availability and downstream signaling (Wang et al., 2024; Baxter, 2023). Emerging evidence also suggests that IGF signaling exerts compartment-specific actions on OA-associated pain. For instance, IGF-1/IGF-1R signaling can increase nociceptor excitability in peripheral inflammatory tissue, whereas in the brain and spinal cord, it inhibits neuroinflammation and PI3K–AKT signaling (Ma et al., 2023; Miura et al., 2011). This suggests IGF signaling may modulate pain through both peripheral sensitization and central neuroimmune mechanisms. Sex differences in both IGF biology and OA-related pain expression further support sex-stratified analyses of IGF–pain associations (Segal et al., 2024).

Epigenetic mechanisms offer an avenue for examining the bidirectional crosstalk between IGF signaling and OA-related joint processes. Genome-wide DNA methylation analyses have identified disease-associated alterations in cartilage and subchondral bone (Zhai and Huang, 2024; Jeffries et al., 2016; Zhang et al., 2016). Several genes within the IGF pathways are epigenetically regulated: *IGF1* is susceptible to methylation, while *IGF2* is regulated through genomic imprinting and methylation at the H19/ICR locus (Ferguson-Smith, 2011; Nordin et al., 2014; Selenou et al., 2022).

DNA methylation alterations are implicated in musculoskeletal and painful disorders through their effects on gene transcription (Liang et al., 2015; Xiong et al., 2024). These alterations may potentially contribute to OA progression (Zhai and Huang, 2024; Knights et al., 2023). One plausible mechanism is that methylation at loci involved in growth, repair, and inflammatory signaling may link aging-related molecular pathways with OA symptoms (Cai et al., 2022; Nau et al., 2025). The IGF pathway is particularly relevant in this context, as both IGF-1 and IGF-2 may mediate OA-related pain (Uchimura et al., 2015; Escribano-Nunez et al., 2024; Roberts et al., 2025). However, the relationship between IGF signaling, epigenetic regulation, and pain severity in human knee OA remains poorly defined. Therefore, the present study examined associations between plasma levels and peripheral DNA methylation of IGF-1 and IGF-2 across radiographic OA–pain phenotypes in middle-aged and older adults, with attention to sex as an effect modifier.

## 2 Methods

### 2.1 Study design

We conducted a cross-sectional analysis of data from adults aged 45 to 85 years with and without knee pain as part of a multi-site study at the University of Florida (UF; Gainesville, Florida, USA) and the University of Alabama at Birmingham (UAB; Birmingham, Alabama, USA). Participants were recruited from the local communities surrounding both institutions through clinic-based and community-based strategies. Individuals were excluded if they had any of the following: rheumatic disorders (such as rheumatoid arthritis or fibromyalgia), prior surgery on the index knee (i.e., the most painful knee), uncontrolled hypertension, neurological disorders, cardiovascular disease, peripheral neuropathy, pregnancy or lactation, pain at another body site more severe than pain in the index knee, chronic opioid therapy, psychiatric hospitalization within the previous year, or impaired cognitive function. The present analysis includes a subset of participants with available data on a panel of senescence biomarkers. The Institutional Review Boards approved the study protocol at both sites, and all participants provided written informed consent before enrollment.

### 2.2 Assessments

#### 2.2.1 Clinical pain

Pain impact was assessed using a knee-specific adaptation of the Graded Chronic Pain Scale (GCPS) (Von Korff et al., 1992). This 7-item self-report instrument evaluates pain intensity and pain-related interference during the previous 6 months. Based on GCPS scoring, participants were categorized as having no pain (grade 0), low-impact chronic pain (grades 1–2), or high-impact chronic pain (grades 3–4). Participants completed the Western Ontario and McMaster Universities Osteoarthritis Index (WOMAC) to assess knee pain, stiffness, and physical function limitations. The total WOMAC score (range 0–96) was derived as the sum of the three subscales: pain (range 0–20), stiffness (range 0–8), and physical function (range 0–68), with higher scores indicating worse knee OA symptoms (McConnell et al., 2001).

#### 2.2.2 Radiographic osteoarthritis (ROA) classification

All participants underwent knee radiography of the index knee using posterior-anterior and lateral views. Images were graded for OA by a board-certified rheumatologist (RS) who was blinded to study participants using the Kellgren-Lawrence (KL) grading system: no OA (grade 0), doubtful OA (grade 1), mild OA (grade 2), moderate OA (grade 3), and severe OA (grade 4) (Kohn et al., 2016). For this analysis, KL grades were grouped into three categories: no ROA (grade 0), early ROA (grades 1–2), and late-stage ROA (grades 3–4).

#### 2.2.3 OA-Pain phenotypes

**Fig. 1:**
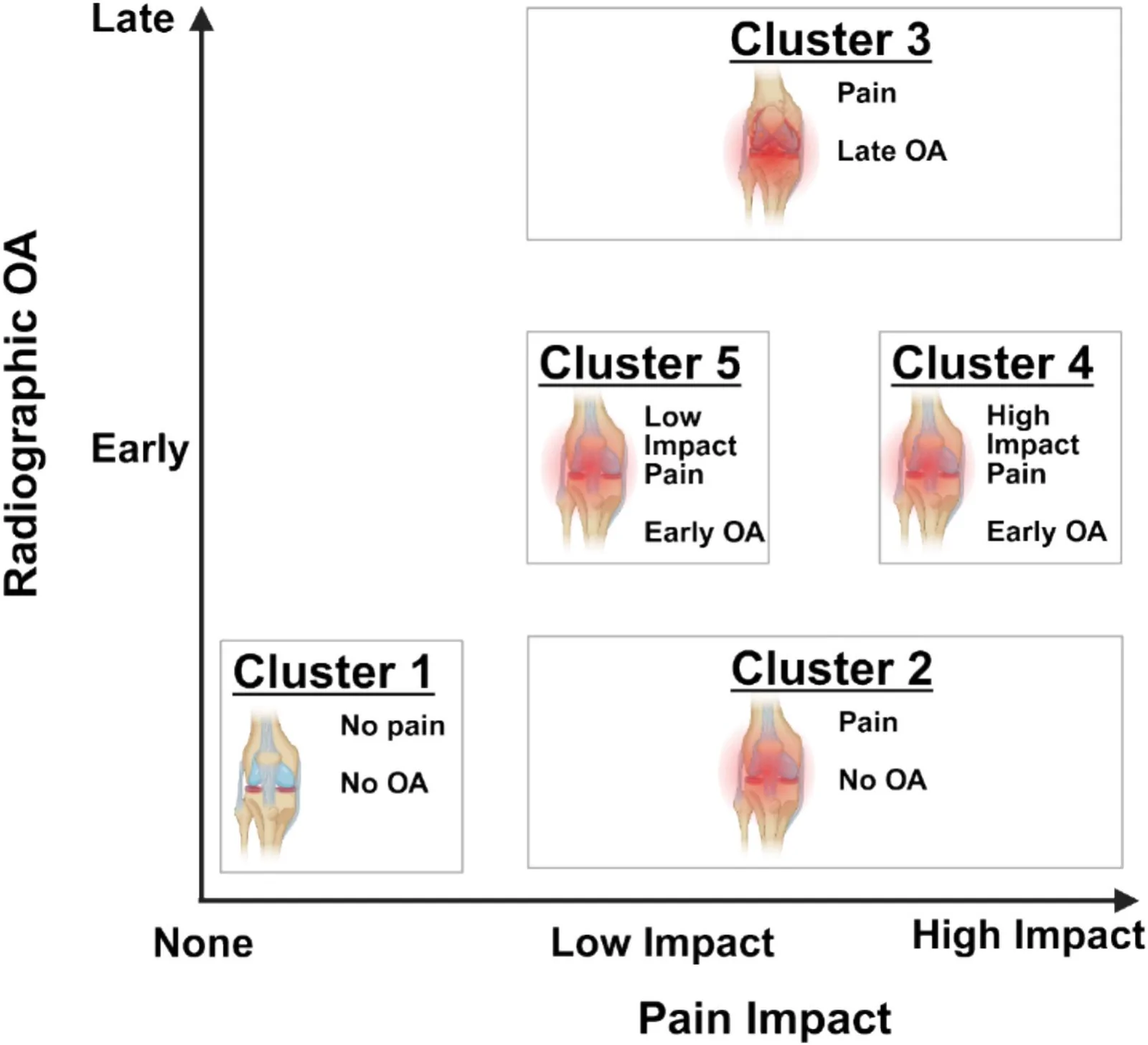
ROA-pain phenotype clusters based on self-reported pain impact and KL grade (Abbas et al., 2026).

Participants were classified using ROA-pain phenotypic groups that were empirically determined in our earlier work (Abbas et al., 2026). Briefly, hierarchical cluster analysis was used to derive clusters based on self-reported pain impact and KL grade, after excluding control participants without pain and without radiographic knee OA. Participants with no pain/no ROA (Cluster 1, *n* = 12) were retained as a separate reference group. The resulting ROA-pain phenotypes are as follows: pain/no ROA (Cluster 2, *n* = 49), pain/late-stage ROA (Cluster 3, *n* = 44), high-impact pain/early-stage ROA (Cluster 4, *n* = 20), and low-impact pain/early-stage ROA (Cluster 5, *n* = 33), as shown in Fig. 1.

#### 2.2.4 Plasma IGF

Venous blood samples were collected into EDTA tubes, gently inverted 8 to 10 times, and kept on ice for less than 30 min before processing. Samples were then centrifuged at 2300 rpm for 15 min at 4 °C, returned to ice immediately afterward, and aliquoted into 2 mL storage vials (Fisher Scientific, Cat# 12567502). Plasma specimens were collected and immediately stored at − 80 °C. The concentrations of Insulin-like Growth Factor 1 (IGF-1) and Insulin-like Growth Factor 2 (IGF-2) in EDTA-plasma samples were determined using a MILLIPLEX® Analyzer 3.1 xPONENT System (Luminex® 200™) and analyzed by the MILLIPLEX® Analyst software: IGF-1, IGF-2 (Millipore; MILLIPLEX MAP Human IGF—I, II Magnetic Bead Panel, Cat#: HIGFMAG-52 K). All assays were performed according to the manufacturer's instructions.

#### 2.2.5 DNA extraction and methylation analysis

EDTA blood tubes were centrifuged at 3000 rpm for 10 min, after which the buffy coat was carefully collected and transferred to cryovials for storage at −80 °C. For genomic DNA extraction, frozen buffy coat samples were thawed at 37 °C until fully homogenized. Approximately 150–200 μL of sample was lysed using red blood cell (RBC) lysis buffer and centrifuged at 6000 rpm for 5 min at room temperature. After removal of the supernatant, sodium EDTA solution was added to the pellet and gently vortexed to disperse residual RBC clumps. The homogenized sample was then incubated at 50–55 °C with Proteinase K and SDS. Following incubation, an equal volume of phenol was added, and the mixture was centrifuged at 10,000 rpm for 10 min. The aqueous phase was transferred to a new tube and subjected sequentially to phenol-chloroform-isoamyl alcohol extraction, followed by chloroform–isoamyl alcohol extraction, with centrifugation at the same speed and duration after each step. DNA was precipitated from the final supernatant by adding one-tenth volume of 3 M sodium acetate and two volumes of absolute ethanol. The DNA pellet was washed with 70% ethanol, centrifuged at 10,000 rpm for 5 min, air-dried, and resuspended in Tris-EDTA buffer. DNA concentration was quantified using Qubit, and quality was assessed by agarose gel electrophoresis. Sodium bisulfite conversion and methylation profiling with the Illumina EPIC array were carried out at the Molecular Genomics Core at Moffitt Cancer Center (3011 Holly Dr., Tampa, FL 33612).

DNA methylation preprocessing and quality control were conducted in R using the *minfi* package. Illumina HumanMethylationEPIC array annotation based on hg19 was used for genomic alignment. Functional normalization was applied to reduce between-array technical variation and to account for variability captured by control probes. Among the initial 866,091 CpG probes, 232 probes could not be mapped to the reference genome, leaving 865,859 mapped probes. We subsequently identified 1275 probes with detection *p*-values >0.01 in more than 10% of samples, 30,064 probes containing a single-nucleotide polymorphism (SNP) at either the CpG interrogation or single-base extension site, and 18,920 probes located on the sex chromosomes. After accounting for overlap among these filtering categories, 50,226 unique probes were removed, leaving 815,633 CpG probes for downstream analyses.

### 2.3 Statistical analysis

Participant characteristics are presented as means and standard deviations for continuous variables and frequencies with percentages for categorical variables, as appropriate. Differences across ROA-pain phenotypes in demographic and pain-related characteristics were evaluated using chi-square tests for categorical variables and analysis of variance (ANOVA) for continuous variables. Post hoc pairwise comparisons were performed with Bonferroni adjustment when appropriate.

To examine the independent associations of pain impact and KL grade with circulating IGF factors, separate unadjusted ANOVA models were conducted for plasma IGF-1 and IGF-2. KL grade was modeled as an ordinal numeric variable in these models. Associations of ROA-pain phenotypes with plasma IGF-1 and IGF-2 levels were then examined using both unadjusted and covariate-adjusted general linear models. Adjusted models included ROA-pain phenotype, sex, and the phenotype-by-sex interaction term, with age, race, body mass index (BMI), and study site entered as covariates. When significant interaction effects were identified, Bonferroni-adjusted simple-effects comparisons were conducted using estimated marginal means.

To identify differential methylation at *IGF1-* and *IGF2-*annotated CpG probes, empirical Bayes–moderated linear models were fitted using the *limma* package. For each CpG probe, methylation level was modeled as the outcome and binary pain clusters (C1 vs. non—C1) as the primary predictor, adjusting for age, sex, race, BMI, and study site. Multiple testing was controlled using the Benjamini–Hochberg false discovery rate (FDR) method.

Putative differentially methylated probes (DMPs) were functionally annotated using the *GenomicFeatures* package in R. Annotation included gene symbol and genomic context, including promoter, exon, intron, and intergenic regions. The proportions of hypermethylated, hypomethylated, and non-significant CpG sites across genomic features were summarized descriptively.

Mean DNA methylation levels for *IGF1* and *IGF2* were calculated by averaging β-values across all CpG probes annotated to each gene. Associations between ROA-pain phenotype and mean methylation levels were examined using the same modeling framework applied to plasma IGF analyses, including unadjusted models and covariate-adjusted models with sex, the phenotype-by-sex interaction, age, race, BMI, and study site. Bonferroni-adjusted estimated marginal means were used for post hoc pairwise and simple-effects comparisons, as appropriate. For all analyses, statistical significance was defined at an alpha level of 0.05 unless otherwise specified.

### 2.4 Human DRG cell atlas

We assessed the Human Dorsal Root Ganglion (DRG) Cell Atlas (Bhuiyan et al., 2025) for expression of IGF ligands *(IGF1, IGF2)* and their receptors *(IGF1R, IGF2R)* across 22 annotated human DRG neuronal subtypes, including those associated with nociception, mechanosensation, thermosensation, and proprioception. No statistical test was performed.

## 3 Results

Participant characteristics compared across pain-ROA clusters are shown in Table 1. Participants had a mean age of 58.1 ± 7.7 (mean ± SD), 91 (57.6%) were female, and 87 (55.1%) identified as non-Hispanic Black. The mean BMI was 31.6 ± 6.9 kg/m^2^. In total, 37.3% of participants (*n* = 59) reported high-impact chronic knee pain, and 55.1% reported low-impact chronic pain (*n* = 87). Similarly, 53 (33.5%) participants had early-stage OA and 44 (27.8%) had late-stage OA.

**Table 1:** Participant characteristics.^a^, ^b^

	Total	Cluster 1 (No pain/ No ROA)	Cluster 2 (Pain/ No ROA)	Cluster 3 (Pain/ Late ROA)	Cluster 4 (High-impact pain/ Early ROA)	Cluster 5 (Low-impact pain/ Early ROA)	
	Mean ± SD n (%)	Mean ± SD n (%)	Mean ± SD n (%)	Mean ± SD n (%)	Mean ± SD n (%)	Mean ± SD n (%)	Probability
**N (%)**	158	12 (7.6)	49 (31.0)	44 (27.8)	20 (12.7)	33 (20.9)	
**Age (years)**	58.1 ± 7.7	58.8 ± 9.6	57.3 ± 7.0	60.1 ± 8.4	57.4 ± 7.8	56.6 ± 6.9	0.310ᵃ
**Sex**							0.317ᵇ
Male	67 (42.4)	4 (33.3)	23 (46.9)	17 (38.6)	12 (60.0)	11 (33.3)	
Female	91 (57.6)	8 (66.7)	26 (53.1)	27 (61.4)	8 (40.0)	22 (66.7)	
**Race**							0.380ᵇ
Non-Hispanic Black	87 (55.1)	9 (75.0)	28 (57.1)	21 (47.7)	9 (45.0)	20 (60.6)	
Non-Hispanic White	71 (44.9)	3 (25.0)	21 (42.9)	23 (52.3)	11 (55.0)	13 (39.4)	
**BMI (kg/m**^**2**^**)**	31.6 ± 6.9	28.3 ± 5.6	28.8 ± 5.5	35.7 ± 7.6	30.9 ± 5.4	32.0 ± 6.4	<0.001ᵃ
**WOMAC**							
Pain	6.3 ± 4.7	0.2 ± 0.6	5.7 ± 4.5	8.2 ± 4.3	9.2 ± 4.5	5.2 ± 4.0	<0.001ᵃ
Stiffness	2.8 ± 2.2	0.2 ± 0.6	2.5 ± 2.2	3.9 ± 1.9	3.7 ± 2.1	2.1 ± 1.6	<0.001ᵃ
Physical function	20.4 ± 15.6	0.9 ± 2.3	18.5 ± 14.7	28.5 ± 15.0	28.3 ± 13.5	15.3 ± 12.4	<0.001ᵃ
Total	29.5 ± 22.0	1.3 ± 3.5	26.7 ± 20.9	40.3 ± 20.5	41.1 ± 19.4	22.4 ± 17.6	<0.001ᵃ
**Study Site**							<0.001ᵇ
UF	112 (70.9)	12 (100.0)	49 (100.0)	25 (56.8)	8 (40.0)	18 (54.5)	
UAB	46 (29.1)	0 (0.0)	0 (0.0)	19 (43.2)	12 (60.0)	15 (45.5)	

Data are presented as mean ± SD or n (%). Percentages in the N (%) row represent the proportion of the total study sample. Percentages for categorical characteristics are calculated within columns.

a ANOVA.

b Chi-square.

The pain-ROA clusters were comparable on age, sex, and race/ethnicity (all *p* > 0.05). On the other hand, BMI varied among clusters (*p* < 0.01), with participants in cluster 3 (pain/late ROA) having the highest BMI (35.7 ± 7.6 kg/m^2^), while those in cluster 1 (no pain/no ROA) had a lower BMI (28.3 ± 5.6 kg/m^2^). Differences among clusters were also observed across WOMAC total scores and their respective pain, stiffness, and physical function subscales (all *p* < 0.01). Specifically, WOMAC total was highest in participants from Cluster 4 (high-impact pain/early OA; 41.1 ± 19.4) and lowest in Cluster 1 (no pain/no ROA; 1.3 ± 3.5).

Table 1 **and** Fig. 1 depict the ROA-pain groups represented by individuals in cluster 1 (no pain/no ROA), cluster 2 (pain/no ROA), cluster 3 (pain/late ROA), cluster 4 (high-impact pain/early ROA), and cluster 5 (low-impact pain/early ROA).

Table 1: Participant characteristics**.**

### 3.1 Associations of pain impact and radiographic OA with circulating IGF factors

To show how pain impact and KL grade are each independently related to IGF factors, we performed univariate analyses using ANOVA. A significant association of KL grade alone with plasma IGF-1 levels (F(1,155) = 7.74, *p* = 0.006) was found, but there was no significant association of pain impact or KL grade alone with plasma IGF-2 levels. Similarly, in univariate ANOVA models, KL grade was significantly associated with *IGF1* methylation levels (F(1,155) = 9.83, *p* = 0.0021), whereas no significant associations were observed between pain impact or KL grade and *IGF2* methylation levels.

### 3.2 Plasma levels of IGF-1 and IGF-2

**Fig. 2:**
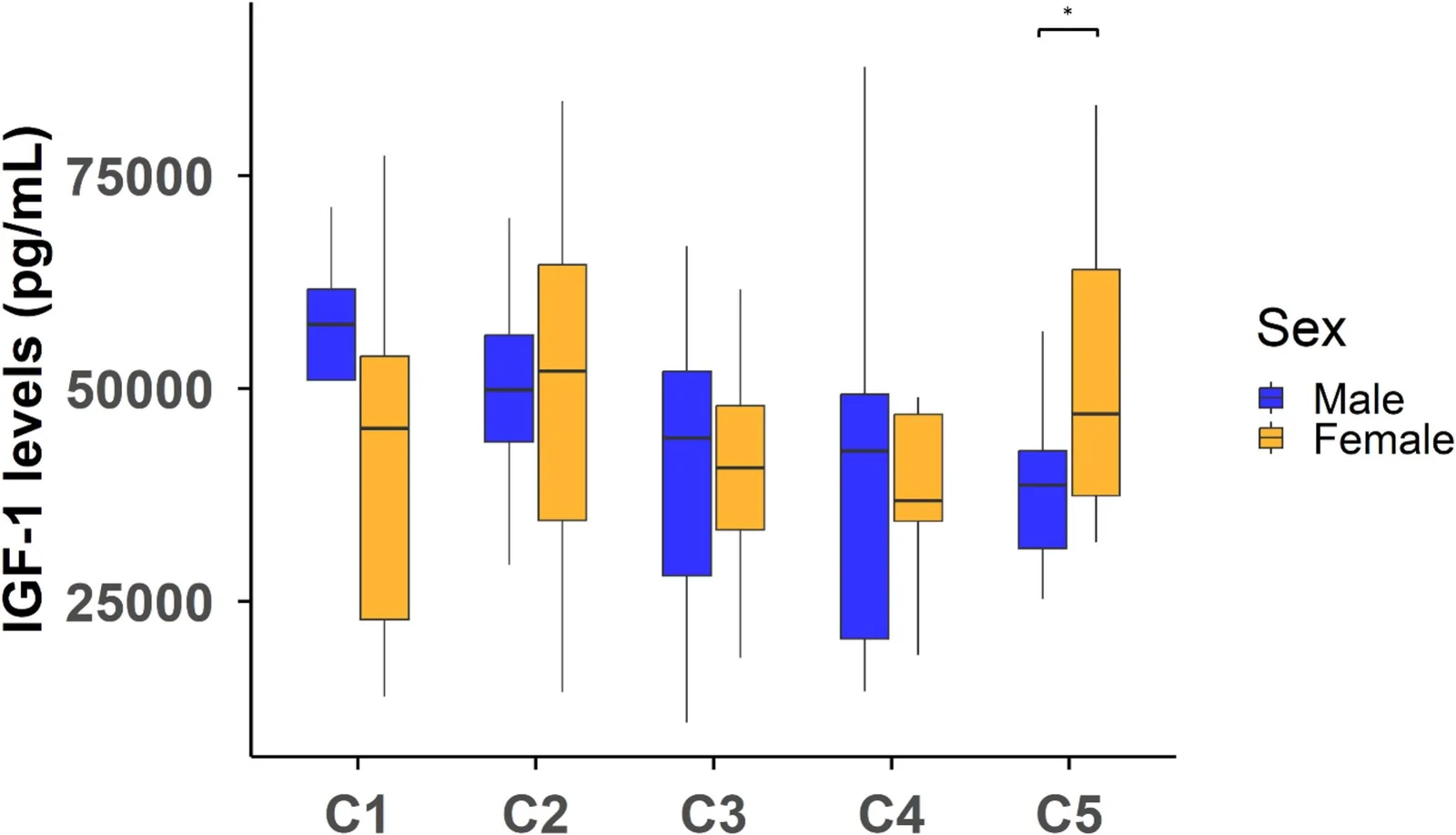
**Association between plasma IGF-1 levels and ROA-pain phenotypes.** The x-axis includes individuals in cluster 1 (no pain/no ROA), cluster 2 (pain/no ROA), cluster 3 (pain/late ROA), cluster 4 (high-impact pain/early ROA), and cluster 5 (low-impact pain/early ROA). The y-axis indicates the corresponding plasma IGF-1 levels.

In the unadjusted model, a significant association was found between ROA-pain phenotypes and IGF-1 plasma levels (F(4,153) = 3.04, *p* = 0.019). After adjustment for covariates, differences in IGF-1 plasma levels between ROA-pain phenotypes were still observed (F(4,144) = 2.61, *p* = 0.038). In Fig. 2**,** post hoc pairwise comparisons were performed between groups, with *p*-values adjusted using the Bonferroni method to identify specific group differences. Females in cluster 5 (early ROA/low-impact pain) had significantly higher IGF-1 levels compared to males (Bonferroni-corrected *p* = 0.0303). No significant associations between ROA-pain clusters and IGF-1 levels were observed among other participants (all p's > 0.05).

**Fig. 3:**
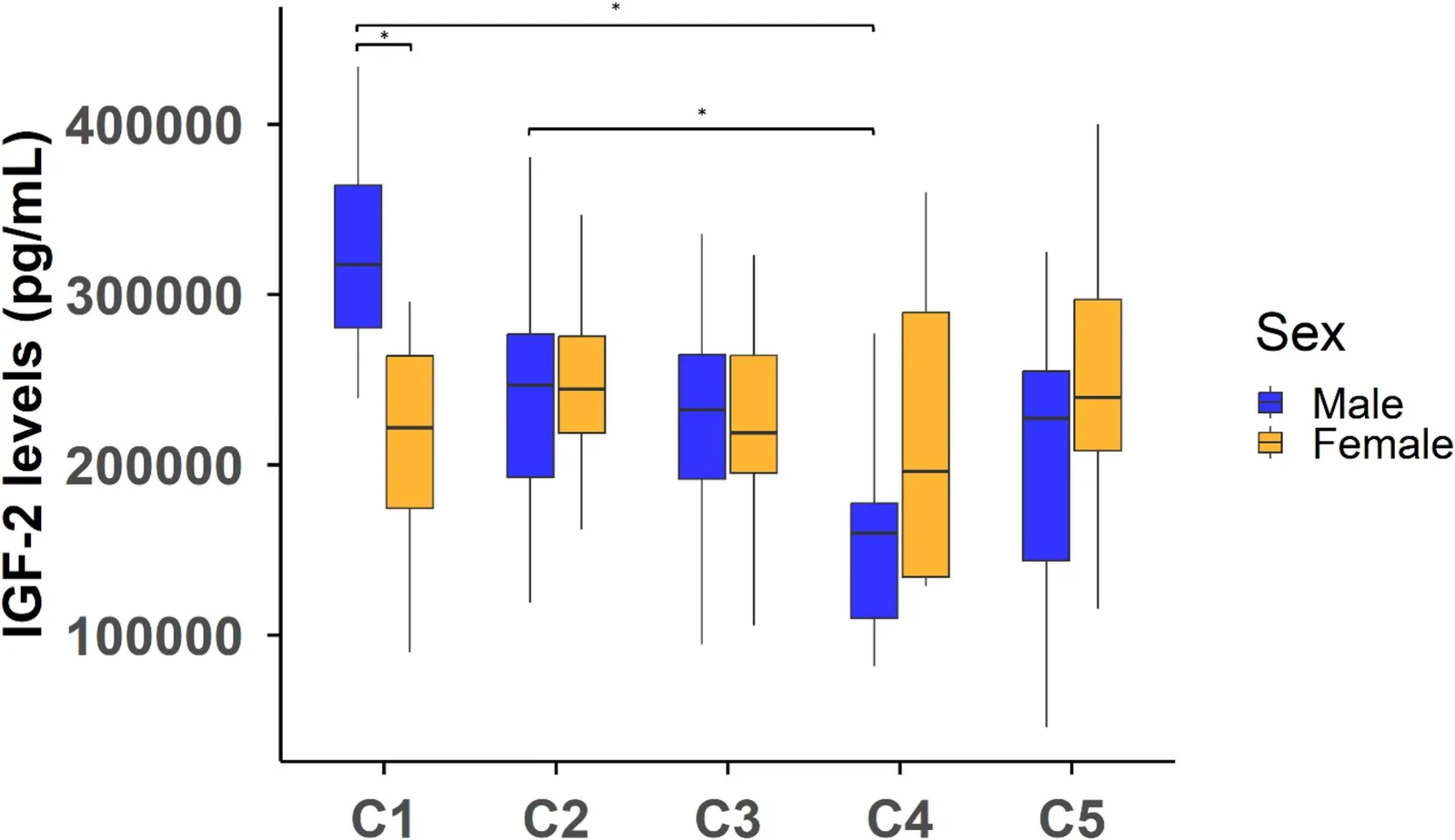
**Association between plasma IGF-2 levels and ROA-pain phenotypes.** The x-axis includes individuals in cluster 1 (no pain/no ROA), cluster 2 (pain/no ROA), cluster 3 (pain/late ROA), cluster 4 (high-impact pain/early ROA), and cluster 5 (low-impact pain/early ROA). The y-axis indicates the corresponding plasma IGF-2 levels. * (*p* < 0.05).

Similarly, in the unadjusted model, a significant association was found between ROA-pain phenotypes and IGF-2 plasma levels (F(4,153) = 2.44, *p* = 0.0496). After adjustment for covariates, differences in IGF-2 plasma levels between ROA-pain phenotypes and sex were observed (F(4,144) = 4.37, *p* = 0.002, ROA-pain phenotypes; F(1,144) = 4.24, *p* = 0.041, sex). In Fig. 3, post hoc pairwise comparisons were performed between groups, with *p*-values adjusted using the Bonferroni method to identify specific group differences. Males in cluster 1 (no pain/no ROA) had significantly higher IGF-2 levels compared to females (Bonferroni-corrected *p* = 0.0413). Males in cluster 1 (no Pain/No ROA) had significantly higher circulating IGF-2 levels compared to males in cluster 4 (Bonferroni-corrected *p* = 0.002). Similarly, males in cluster 2 had significantly higher circulating IGF-2 levels compared to males with high-impact pain and early ROA in cluster 4 (Bonferroni-corrected *p* = 0.0353). No significant associations between ROA-pain clusters and IGF-2 levels were observed among other participants (all p's > 0.05).

### 3.3 IGF-related CpGs associated with pain

IGF-related CpGs were assessed for association with binary pain clusters (C1 vs. non—C1) using a model adjusted for age, race, site, sex, and BMI. However, no IGF CpG sites were significant after FDR correction (*p* < 0.05). At a raw *p* < 0.05 cutoff, 5 out of 29 *IGF1* CpG probes showed associations (3 hypermethylated and 2 hypomethylated), distributed across intron, intergenic, and promoter regions within Open Sea (>4 kb away from nearest CpG island). Similarly, 6 out of 121 *IGF2* CpG probes showed associations (3 hypermethylated and 3 hypomethylated), distributed across exon, intron, and promoter regions within N_Shelf (2–4 kb from a CpG island), S_Shore (0–2 kb), and N_Shore (0–2 kb) regions (Table 2 **and full list in Tables S1 and S2**). None of these associations remained significant after multiple testing correction (q = 0.28–0.39).

**Table 2:** IGF CpG sites associated with pain.

ID	Estimate	SE	CI_lower	CI_upper	pval	qval	Genome	chr	start	end	CpG_Island	Feature Annotation	Gene	Direction
cg12128490	−0.23092	0.084045	−0.39696	−0.06488	0.006828	0.284754	37	12	102,878,335	102,878,335	Sea	Intergenic	*IGF1*	Hypo
cg21750689	−0.37032	0.152066	−0.67074	−0.0699	0.014935	0.324586	37	12	102,874,649	102,874,649	Sea	Promoters	*IGF1*	Hypo
cg00534542	0.175225	0.078349	0.020439	0.330011	0.027479	0.35862	37	12	102,835,223	102,835,223	Sea	Introns	*IGF1*	Hyper
cg07020773	0.29756	0.139539	0.021888	0.573233	0.032863	0.370682	37	12	102,873,260	102,873,260	Sea	Promoters	*IGF1*	Hyper
cg12742178	0.314016	0.15659	0.004658	0.623374	0.044325	0.392698	37	12	102,815,929	102,815,929	Sea	introns	*IGF1*	Hyper
cg22932993	0.15744	0.063275	0.032434	0.282446	0.015431	0.326524	37	11	2,156,609	2,156,609	N_Shelf	Exons	*INS-IGF2;IGF2;MIR483*	Hyper
cg23889607	−0.22099	0.100065	−0.41868	−0.0233	0.028167	0.360284	37	11	2,150,902	2,150,902	N_Shelf	Exons	*INS-IGF2;IGF2;MIR483*	Hypo
cg02808220	0.123519	0.057166	0.010583	0.236455	0.036284	0.377202	37	11	2,156,282	2,156,282	S_Shore	Promoters	*INS-IGF2;IGF2;MIR483*	Hyper
cg05075097	−0.21214	0.104981	−0.41954	−0.00474	0.044093	0.392306	37	11	2,173,341	2,173,341	N_Shelf	Introns	*INS-IGF2;IGF2;IGF2-AS*	Hypo
cg10501065	−0.20074	0.099233	−0.39678	−0.0047	0.04416	0.39243	37	11	2,164,976	2,164,976	N_Shore	Introns	*INS-IGF2;IGF2;IGF2-AS*	Hypo
cg06029905	0.140515	0.068754	0.004686	0.276345	0.044964	0.393783	37	11	2,155,521	2,155,521	S_Shore	Promoters	*INS-IGF2;IGF2;MIR483*	Hyper

Table 2 **: IGF CpG sites associated with pain.**

### 3.4 Peripheral Methylation levels of *IGF1* and *IGF2*

**Fig. 4:**
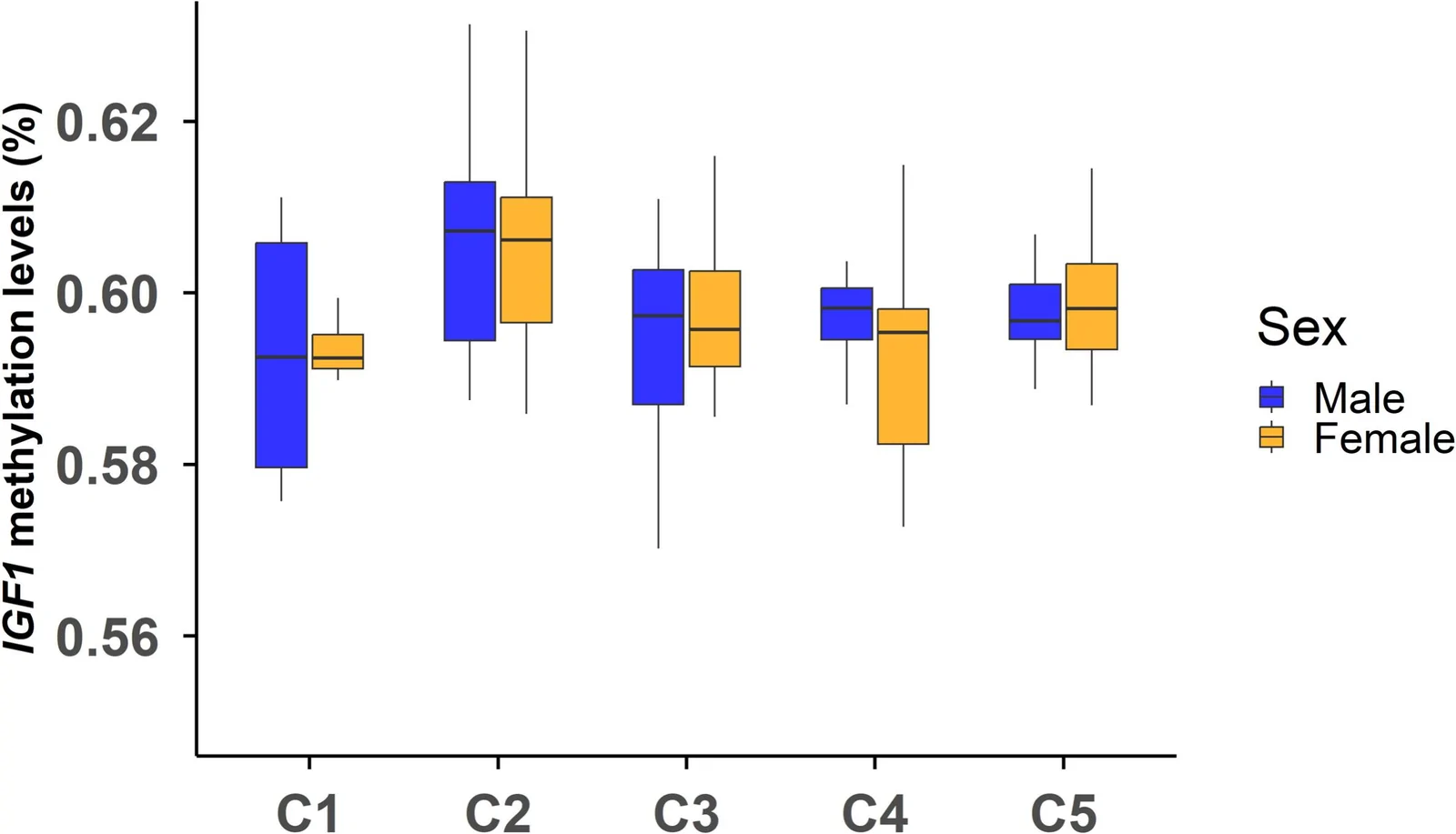
**Association between average *IGF1* methylation levels and ROA-pain phenotypes.** The x-axis includes individuals in cluster 1 (no pain/no ROA), cluster 2 (pain/no ROA), cluster 3 (pain/late ROA), cluster 4 (high-impact pain/early ROA), and cluster 5 (low-impact pain/early ROA). The y-axis indicates the corresponding average *IGF1* methylation levels.

In the unadjusted model, a significant association was found between ROA-pain phenotypes and *IGF1* methylation levels (F(4,153) = 3.69, *p* = 0.007). However, in the adjusted model, no significant associations were observed between *IGF1* methylation and sex, ROA-pain phenotypes, or their interaction (all p's > 0.05; Fig. 4).

**Fig. 5:**
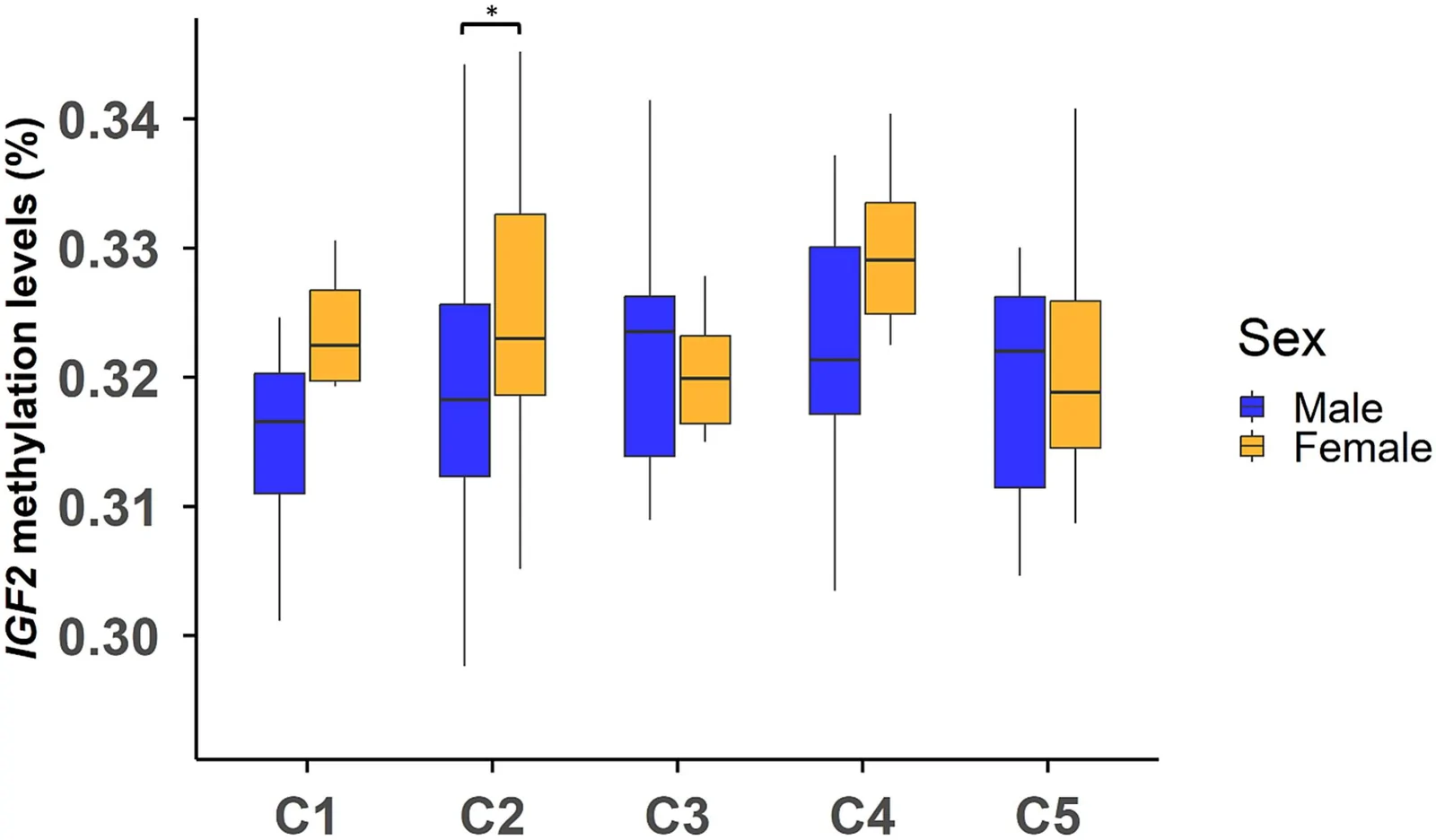
**Association between OA-pain phenotypes with average *IGF2* methylation levels.** The x-axis includes individuals in cluster 1 (no pain/no ROA), cluster 2 (pain/no ROA), cluster 3 (pain/late ROA), cluster 4 (high-impact pain/early ROA), and cluster 5 (low-impact pain/early ROA). The y-axis indicates the corresponding average *IGF2* methylation levels.

In the unadjusted model, there was no overall association found between ROA-pain phenotypes and *IGF2* methylation levels. However, in the adjusted model, post-hoc simple-effects tests indicated that within cluster 2, females had higher *IGF2* methylation levels than males (Bonferroni-corrected *p* = 0.037, Fig. 5).

### 3.5 IGF ligand and receptor expression in human DRG neuronal subtypes

*IGF1R* and *IGF2R* were expressed across all human DRG neuronal subtypes, whereas *IGF1* and *IGF2* showed consistently low expression across all subtypes (Fig. S1).

## 4 Discussion

Dysregulation of the insulin-like growth factors (IGF) has been implicated in osteoarthritis (OA), including articular cartilage degeneration, chondrocyte senescence, and joint inflammation (Uchimura et al., 2015; Yin et al., 2009; Loeser, 2009; Fortier et al., 2011) but their role in OA-related pain remains unclear. Large-scale genome-wide association studies (GWAS) have identified genetic loci associated with knee OA and pain (Meng et al., 2019; Hatzikotoulas et al., 2025), but these studies have emphasized genetic risk rather than circulating biomarkers of OA-related pain. In this study, we evaluated plasma levels and DNA methylation signatures of IGF-1 and IGF-2 in relation to chronic knee OA pain among middle-aged to older adults, across distinct OA-pain phenotypes defined by pain impact and the structural severity of radiographic knee OA. We observed sex-dependent associations between OA-pain phenotypes and circulating growth factors, particularly IGF-2. Across the five ROA–pain phenotypes, plasma IGF-1 and IGF-2 levels were associated with phenotype in a sex-dependent manner, most notably, males with high-impact pain and early ROA had significantly lower IGF-2 levels than males without pain or radiographic disease, whereas peripheral DNA methylation of *IGF1* and *IGF2* showed only limited associations after covariate adjustment, with no CpG probe surviving FDR correction.

### 4.1 Sex-modified circulating IGF-1 levels

Our findings showed that females with early ROA and low-impact pain had significantly higher IGF-1 levels compared to their male counterparts. Previous studies have detailed the protective role of IGF-1 in OA by demonstrating that it prevents chondrocyte and matrix degradation in animal models by increasing the production of collagen and proteoglycans (Wei et al., 2017; Collins et al., 2023). Ekenstedt et al. have demonstrated that reduced levels of IGF-1 increase articular cartilage degeneration in OA, without the bony lesions typically seen in this disease (Ekenstedt et al., 2006). Escribano-Nuñez et al. have reported that *IGF1* is a target gene of the Wnt pathway in articular chondrocytes (Escribano-Nunez et al., 2024). The study also reports that the upregulation of *IGF1* following Wnt activation is associated with OA disease progression (Escribano-Nunez et al., 2024). However, our findings were only significant within individuals reporting low-impact pain with early ROA. These findings may suggest a potential role for IGF-1 in modulating OA pain pathways.

IGF-1 may have dual roles in cartilage protection and pain modulation. In a systematic review, Claessen et al. found moderate evidence of no association between serum IGF-1 and radiographic OA after adjusting for age, sex, and BMI (Claessen et al., 2012). Our findings suggest that pain should also be considered in this relationship. Moreover, Ma et al. reported that IGF/IGF-1R signaling plays compartment-specific roles in pain modulation (Ma et al., 2023). Peripherally, the IGF/IGF-1R pathway is primarily associated with pain promotion, whereas centrally, in the brain and spinal cord, it inhibits neuronal excitation and reduces pain (Ma et al., 2023; Miura et al., 2011). The actions of IGF-1/IGF-1R signaling on the nervous system include glial regulation, neuroinflammation, autophagy, demyelination, and PI3K/AKT signaling (Ma et al., 2023). As shown by the Human DRG Cell Atlas, *IGF1R* and *IGF2R* are expressed across all annotated human DRG neuronal subtypes, whereas *IGF1* and *IGF2* are expressed at much lower levels (Fig. S1) (Bhuiyan et al., 2025). DRGs are present outside the blood-brain barrier (Haberberger et al., 2019), and show significantly greater permeability and interstitial leakage than adjacent spinal nerves measured in humans in vivo (Godel et al., 2016). Therefore, circulating IGFs may reach sensory neuron somata. However, IGF activity is modulated by IGFBPs, which regulate ligand availability and downstream signaling (Wang et al., 2024; Baxter, 2023). In preclinical models, IGF-1R has been implicated in nociceptor sensitization via PI3K/AKT signaling in rat DRG (Miura et al., 2011), and Zhang et al. reported that IGF-1 enhances the sensitivity of sensory neurons to noxious stimuli. IGF-1 increased the activity of the voltage-gated calcium channel Ca_V_3.2 in mouse sensory neurons (Zhang et al., 2014). In contrast, IGF-2R contributes to lysosomal enzyme transport and endocytosis-mediated ligand clearance in the spinal cord and DRG of the rat (Hawkes and Kar, 2002), but its role in nociceptive signaling has not been well defined.

### 4.2 Plasma IGF-2 and high-impact pain in males

In contrast, among those with no ROA and no pain (i.e., controls), males had higher IGF-2 levels than females. Within males, no ROA-no pain controls, as well as those with pain but no ROA, had higher plasma IGF-2 levels compared to those with early OA and high-impact pain. To our knowledge, this is the first study to show a potential novel association between IGF-2 and high-impact chronic knee pain associated with OA, as prior studies assessing the association between IGF-2 and knee OA pain are limited. Previous studies have indicated that IGF-2 plays a pivotal role in halting joint degradation in OA, preventing the formation of osteophytes and subchondral bone sclerosis and promoting cartilage integrity (Uchimura et al., 2015). Uchimura et al. demonstrated that IGF-2 inhibits IL-1β-induced cartilage matrix loss and promotes cartilage integrity in experimental OA (Uchimura et al., 2015). Overall, these studies suggest that IGF-2 may play a protective role in cartilage, which could be relevant for pain modulation. In our study, IGF-2 showed stronger plasma associations with ROA-pain phenotypes than IGF-1, although circulating levels may not reflect local joint or neuronal IGF activity.

### 4.3 Peripheral DNA methylation of IGF-1 and IGF-2

Despite a univariate association between mean *IGF1* methylation and OA structural severity, peripheral DNA methylation effects were largely attenuated after covariate adjustment, and no CpG probe survived FDR correction. Females in cluster 2 (Pain/No ROA) had significantly higher *IGF2* methylation levels than males, demonstrating potential sex-specific epigenetic regulation. *IGF2* methylation is also allele-specific due to imprinting, further limiting interpretability (Ferguson-Smith, 2011). Sex-specific factors, such as hormones (e.g., estrogen and testosterone), may influence IGF levels and warrant investigation in future studies (Veldhuis et al., 2005).

### 4.4 Strengths and limitations

Strengths of this study include the use of empirically derived ROA–pain phenotypes that integrate self-reported pain impact with radiographic severity, the parallel assessment of circulating protein and peripheral DNA methylation for both IGF-1 and IGF-2, and a sex-stratified analytic framework. Several limitations should also be considered. The cross-sectional design precludes causal inference between IGF signaling and OA pain. The no-pain/no-ROA control group was small (*n* = 12), and site composition was uneven, with UAB participants contributing only to clusters C3–C5; this limits the separability of site and phenotype effects, particularly for methylation analyses. Peripheral blood (buffy coat) DNA methylation may not reflect tissue-specific epigenetic regulation in cartilage, synovium, or sensory neural tissues most relevant to OA-related pain. In addition, *IGF2* is regulated through genomic imprinting at the H19/ICR locus, so allele-averaged β-values may obscure parent-of-origin–specific methylation signals. Finally, no CpG probe survived FDR correction; probe-level associations should therefore be regarded as hypothesis-generating and require replication in larger cohorts with longitudinal sampling and tissue-level epigenetic measurement.

## 5 Conclusions

In summary, plasma IGF-1 and IGF-2 levels are associated with OA-pain phenotypes in a sex-dependent manner, with IGF-2 levels higher in males with no pain, and lower in those with high-impact pain, while peripheral DNA methylation showed limited associations with these phenotypes. Females with early ROA/low-impact pain had significantly higher IGF-1 levels compared to males. Among those with pain/no ROA, females had higher *IGF2* methylation levels than males. IGF-2 associations with ROA pain may partly reflect a cartilage-protective mechanism, though cartilage integrity was not evaluated in our study. Future studies should evaluate cartilage integrity using imaging or histological measures. These findings highlight the importance of endocrine and sex-related biological factors in OA-related pain and motivate further investigation into tissue-specific epigenetic regulation of IGF pathways. Future research with larger, longitudinal cohorts is needed to validate and extend these findings.

## CRediT authorship contribution statement

**Muhammad Abbas:** Writing – review & editing, Writing – original draft, Methodology, Investigation, Formal analysis, Conceptualization. **Javier A. Tamargo:** Writing – review & editing, Writing – original draft, Methodology. **Yutao Zhang:** Visualization, Formal analysis. **Carlos J. Cruz:** Writing – review & editing. **John Gilliam:** Writing – review & editing. **Stephanie Wohlgemuth:** Resources, Investigation. **Kevin Wu:** Resources, Investigation. **Roland Staud:** Writing – review & editing, Resources, Investigation. **Li Chen:** Visualization, Validation, Formal analysis. **Burel R. Goodin:** Writing – review & editing, Resources, Project administration, Investigation, Data curation. **Roger B. Fillingim:** Writing – review & editing, Supervision, Resources, Project administration, Investigation, Funding acquisition, Data curation. **Christiaan Leeuwenburgh:** Writing – review & editing, Supervision, Methodology, Investigation. **Yenisel Cruz-Almeida:** Writing – review & editing, Supervision, Resources, Project administration, Methodology, Investigation, Funding acquisition, Formal analysis, Data curation, Conceptualization.

## Ethics declaration

Written informed consent to take part in the study and to publish the article has been obtained from all participants or their legal representatives. The privacy rights of participants have been observed.

This study included organ or tissue donors.

This study includes human biological material and consent was obtained by donors, or their next of kin or legal representatives, for use in this study and for publication of the article. The samples used in this research were not sourced from executed prisoners or prisoners of conscience.

This study was performed in compliance with relevant laws, regulatory frameworks and guidelines where the research took place.

This study was approved by the UF IRB.

(Approval No. IRB201500906)

## Funding

This work was supported by the National Institute of Arthritis and Musculoskeletal and Skin Diseases of the National Institutes of Health under award numbers R90AR085802 and T90AR085527, the 10.13039/100000049National Institute on Aging R01AG059809, R01AG067757, R37AG033906, T32AG049673 and L32AG096730, and the University of Florida Pain Research & Intervention Center of Excellence.

## Declaration of competing interest

The authors declare that they have no known competing financial interests or personal relationships that could have appeared to influence the work reported in this paper.

## Data Availability

Data will be made available on request.

## Appendix A Supplementary data

Supplementary Material 1 Supplementary Table 1: Full list of *IGF1* CpG sites associated with pain.

Supplementary Material 2 Supplementary Table 2: Full list of *IGF2* CpG sites associated with pain.

Supplementary Material 2 Supplementary Fig. 1: *IGF1, IGF2, IGF1R,* and *IGF2R* expression across human DRG neuronal subtypes.
