# Supplementary material for: Plasma levels and peripheral DNA methylation of IGF-1 and IGF-2 across knee osteoarthritis pain phenotypes in middle-aged and older adults
Source: Neurobiol Pain. 2026 Sep 7;20:100233. doi: 10.1016/j.ynpai.2026.100233 (PMC13587716; doi:10.1016/j.ynpai.2026.100233)
Supplement: Supplementary file 1 [file mmc3.docx]

**Fig. S1: *IGF1, IGF2, IGF1R,* and *IGF2R* expression across human DRG neuronal subtypes.** Bubble plot showing expression (color; z-scored) and proportion of cells expressing each gene (dot size) across 22 annotated human DRG neuronal subtypes associated with nociception, mechanosensation, thermosensation, and proprioception. Data source: Human DRG Cell Atlas (<https://painseq.shinyapps.io/u19humandrgatlas/>).^35^
